# Optimization and Characterization of Lupin Protein Isolate Obtained Using Alkaline Solubilization-Isoelectric Precipitation

**DOI:** 10.3390/foods12203875

**Published:** 2023-10-23

**Authors:** Rubén Domínguez, Roberto Bermúdez, Mirian Pateiro, Raquel Lucas-González, José M. Lorenzo

**Affiliations:** 1 Centro Tecnológico de la Carne de Galicia, Rúa Galicia N° 4, Parque Tecnológico de Galicia, 32900 San Cibrao das Viñas, Ourense, Spain; robertobermudez@ceteca.net (R.B.); mirianpateiro@ceteca.net (M.P.); raquel.lucasg@umh.es (R.L.-G.); jmlorenzo@ceteca.net (J.M.L.); 2 IPOA Research Group, Centro de Investigación e Innovación Agroalimentaria y Agroambiental (CIAGRO), Miguel Hernández University, 03202 Elche, Alicante, Spain; 3 Área de Tecnoloxía dos Alimentos, Facultade de Ciencias, Universidade de Vigo, 32004 Vigo, Ourense, Spain

**Keywords:** *Lupinus luteus*, protein isolate, functional properties, chemical composition, amino acids, vegetable protein

## Abstract

The trend in today’s society is to increase the intake of vegetable protein instead of animal protein. Therefore, there is a concern to find new sources of alternative protein. In this sense, legumes are the main protein source of vegetable origin. Of all of them, lupins are the ones with higher protein content, although they are currently undervalued as an alternative for human consumption. In this sense, it is vital to characterize and obtain protein isolates from this legume, which satisfies the growing demand. Therefore, in the present work, the procedure for obtaining a lupin (*Lupinus luteus*) protein isolate (LPI), based on basic solubilization followed by isoelectric precipitation, has been optimized and validated. The optimized LPI, as well as the lupin flour, were subsequently characterized. The chemical composition, physicochemical, as well as the technofunctional properties of the LPI were analyzed. The results show that the proposed procedure had a high yield (23.19 g LPI/100 g flour) and allowed to obtain high-purity protein isolates (87.7 g protein/100 g LPI). The amino acid composition and the chemical scores show high proportions of essential amino acids, being protein deficient only in methionine and valine. Therefore, it can be affirmed that it is a high-quality protein that meets the requirements proposed by the FAO. Regarding the lipid fraction, it is mainly composed of unsaturated fatty acids (C18:1n-9 and C18:2n-6), which is also advisable in order to follow a healthy diet. Finally, LPI showed interesting technofunctional properties (foaming, gelling, emulsifying, water and oil absorption, and solubility), which makes it especially attractive for use in the food industry.

## 1. Introduction

In recent years, there is a growing trend to find alternative protein sources to animal proteins. Legumes are one of the main sources of plant-based protein and have historically been an important protein source for the human diet [1]. Legumes also present other benefits, since they are adapted to a wide range of climatic conditions and fix atmospheric nitrogen. Among them, lupins have a high proportion of proteins and they have emerged as a cheap functional food [2,3]. In fact, protein contents ranged between 30 and 50%, depending on the lupin specie, with an excellent amino acid profile, particularly rich in lysine in contrast to other plant proteins [3]. However, taking into account their potential use in the food industry and in human nutrition, lupins are underused legumes [4]. The most important lupin species include white lupin (*Lupinus albus*), narrow-leaved lupin (*Lupinus angustifolius*), pearl lupin (*Lupinus mutabilis*), and yellow lupin (*Lupinus luteus*) [2]. Moreover, in addition to nutritional aspects, the consumption of lupin proteins was related to hypolipidemic, hypoglycemic, hypotensive, anticarcinogenic, and antiobesity properties [5].

There is increasing interest in the production of protein isolates and concentrates since they are vital for food processing and are used in several applications in the food industry [2,6]. Among lupin species, yellow lupin (*L. luteus*) normally presented the highest protein and the lowest fat contents, which are two essential qualities to produce protein isolates [2]. Generally, two main procedures exist to extract proteins from lupin. These include dry-fractionation, in which proteins are separated from the other constituents according to their size, density, and electrostatic properties [7], and wet-extraction, in which proteins are solubilized at alkaline pH, and they are recovered by precipitation at isoelectric point. Other extractions (wet extractions) consist of micellization (salt extraction) and selective fractionation (acid extractions) [4]. Among both procedures, wet-extraction is better, since protein isolates that are produced have higher purity, digestibility, and quality, while protein concentrates obtained with dry-fractionation presented low purity (~50%) [8], with high amounts of other constituents (carbohydrates, lipids, etc.), and thus should be further processed for concentration [6]. Moreover, among the wet-extraction procedures, alkaline solubilization-isoelectric precipitation is the most used and resulted in LPI with high purity (i.e., in comparison with micellization), but it is important to highlight that this procedure promotes protein denaturation, which can affect the lupin protein isolate’s (LPI) technofunctional properties [9].

Due to the aforementioned advantages, many studies use wet-extraction as the main method to obtain protein isolates from lupin or from other legumes [2]. Most focus on the first stage of solubilization at alkaline pH, followed by a separation of the insoluble fraction, and a precipitation of the proteins at the isoelectric point. There are countless procedures, which include steps in which moderate temperatures are applied, which are left to rest overnight, or undergo applications of different pH (between 8 and 11 for protein solubilization and between 4 and 6 for precipitation). It is well known that temperature can significantly affect the conformation of proteins, producing protein denaturalization and insolubilization, and therefore it could compromise their technological properties [10]. Moreover, the fact of carrying out long procedures, and with stages of rest overnight, means that they are not the most neither suitable nor efficient for the industry, which would make it difficult to be able to scale these protocols. Additionally, some compounds such as oil and carotenoids are present in lupin seeds, and the defatted step is a typical procedure before protein extraction [2]. The defatted procedure increases purity and yield, and also affects the techno-functional properties [6]; however, it is also a previous step that adds complexity to the process and the application of unwanted solvents to the sample. Moreover, the use of some solvents during the defatted phase increased protein denaturation, resulting in decreased solubility and lower protein recovery [4]. Protein extraction is a very complex procedure, which includes the penetration of the solvent into the cells and the correct solubilization of the proteins [11]. Thus, several authors conclude that optimization of the extraction protocol is essential to be able to obtain high yields of lupin protein isolates of high purity [4]. Moreover, the optimum pH for alkali solubilization needs to be explored further to increase yield and LPI quality [9]. For all these reasons, in this study, an extraction based on wet-extraction has been proposed, but without a defatted step, the application of temperature or long stages, which optimize the pH values, allow to obtain the highest yield and highest purity.

Therefore, the main objective of the present study was to design an efficient, fast, simple, and safe process to obtain the lupin protein isolate. For this, a simple protocol was employed and the main extraction conditions, which include solubilization pH, precipitation pH, and extraction time were optimized.

## 2. Materials and Methods

### 2.1. Raw Material

Lupin seeds (*Lupinus luteus* L. [Tremosilla]) were purchased from Semillas Batlle S.A. (Barcelona, Spain). They were ground to obtain homogeneous lupin flour.

### 2.2. Preparation of Lupin Protein Isolate

**Figure 1:**
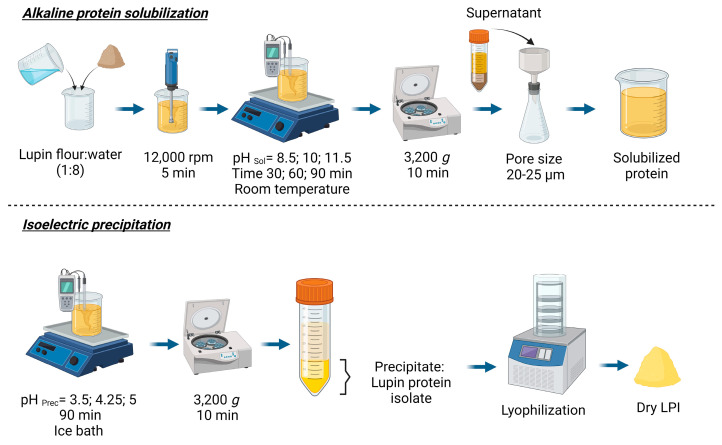
Schematic representation of the LPI preparation process.

The lupin protein isolates (LPI) were prepared from lupin flour (Figure 1), using the pH and time conditions specified in the experimental design section. Briefly, flour was dispersed with distilled water (1:8 *w*/*w*) and homogenized with UltraTurrax (IKA, model T18; Staufen, Germany) for 5 min at 12,000 rpm. The protein was solubilized by adjustment of pH at 8.5–11.5 with 2M NaOH. The mixture was stirred (magnetic stirred) and protein extraction was tested at three different times (30, 60, and 90 min) and at room temperature. Then, the mixture was centrifuged (3200× *g* for 10 min; Beckman Coulter (Brea, CA, USA), model Allegra X-22R, rotor SX4250) to separate the residual starch and insoluble fibers, and the supernatant was filtered through a paper filter (pore size 20–25 µm; Filterlab 1238, Barcelona, Spain). The pellet was washed with distilled water with the pH adjusted at the desired extraction pH, centrifuged, and filtered again. Both supernatants were combined.

For protein precipitation (isoelectric point), the supernatant was placed in an ice bath, the pH was adjusted to pH 3.5–5.0 with 4M HCl and magnetically stirred for 30 min. Then, the mixture was left to rest for an hour. The protein isolate was recovered by centrifugation (3200× *g* for 10 min; Beckman Coulter (Brea, CA, USA), model Allegra X-22R, rotor SX4250), and the LPI was washed with distilled water (1:8 *w*/*v*), and centrifuged again (3200× *g* for 10 min; Beckman Coulter (Brea, CA, USA), model Allegra X-22R, rotor SX4250). The precipitate was lyophilized (Lyovapor L300, Büchi; Barcelona, Spain; Primary drying pressure limit: 0.500 mbar, time 240 min; Secondary drying pressure limit: 0.400 mbar, time 120 min) and stored at −20 °C until further analysis.

### 2.3. Experimental Design and Optimized Responses

This study was conducted using an independent quadratic Box–Behnken experimental design (3-factor and 3-level) with 15 experimental runs and three center points (3 × 1 × 15) (Table 1). The response surface methodology (RSM) was used to identify the optimal levels of the independent variables for maximize the responses. The effect of the solubilization pH (pH _sol_; x_1_), extraction time (x_2_; minutes), and precipitation pH (pH _prec_; x_3_) (independent variables) on the protein extraction yield (y_1_) and LPI purity (y_2_) (dependent variables) were studied. The experimental data were adjusted to the second-order polynomial model, which describes the interaction between the factors and response variables obtained through RSM, according to Equation (1).

(1) $$Y=\beta_{0}+\sum_{i=1}^{3}\beta_{i}X_{i}+\sum_{i=1}^{3}\beta_{ii}X_{i}^{2}+\sum_{i=1}^{3}\times \sum_{j=i+1}^{3}\beta_{ij}X_{i}X_{j}$$

where the *Y* is the predicted result; *β*_0_ is a coefficient of the models; *β_i_*, *β_ii_*, and *β_ij_* are the coefficients of the equations representing linear, quadratic, and interaction models, respectively; and *X_i_* and *X_j_* are the independent variables that determine changes in the response variable.

The adjustment of the model and statistical values was determined using an ANOVA test. The dependent variables were analyzed to obtain the optimal conditions using a multi-response surface optimization, and the optimal extraction conditions were estimated with the response desirability profiling function. For the model validation, four independent extractions were carried out at the optimal conditions, and predicted and experimental values were compared.

### 2.4. Lupin Flour and Optimized LPI Characterization

#### 2.4.1. Chemical Composition and Color Determination

The chemical composition of lupin flour and lyophilized LPI obtained at the optimal conditions was determined according to ISO procedures for protein [12] (N × 6.25) and ash [13], while lipid content was determined according to Procedure Am 5–04 [14]. Color parameters were measured using a portable CR-400 colorimeter (Konica Minolta Sensing Inc., Osaka, Japan).

#### 2.4.2. Fatty Acid Analysis

The fatty acid determination was carried out with gas chromatography. Briefly, lupin oil was extracted using the Bligh and Dyer [15] procedure. Then, fatty acids were trans-esterified with sodium methoxide and sulfuric acid-methanol solutions [16]. The fatty acids methyl esters were separated, identified, and quantified using GC-FID (GC-Agilent 7890B, Agilent Technologies, Santa Clara, CA, USA), equipped with a capillary column DB-23 (60 m, 0.25 mm i.d., 0.25 µm film thickness; Agilent Technologies). The procedure and the chromatographic conditions were previously described by Barros et al. [16]. The fatty acids results were expressed as g/100 g of total fatty acids.

#### 2.4.3. Amino Acid Analysis and Chemical Score

The amino acid content of the lupin flour and LPI was determined using liquid chromatography, following the sample treatment, derivation, and chromatographic conditions described by Munekata et al. [17]. Briefly, the LPI samples (0.1 g) were hydrolyzed with HCl (6N) for 24 h at 110 °C. Then, the extracts were derivatized using the AccQ-Tag method (Waters, Milford, MA, USA), and the separation, identification, and quantification were performed in a high-performance liquid chromatography (Alliance 2695 model, Waters, Milford, MA, USA) using a scanning fluorescence detector (model 2475, Waters). All results were expressed as mg/g protein.

The amino acid composition of LPI was used for the determination of chemical score, considering the values of essential amino acids of the sample (EAAs) and the pattern concentration (EAAp) according to FAO/WHO/UNU [18] for adults:

(2) $$Chemical\;Score\;\left(\%\right)=\frac{Essential\;amino\;acid\;in\;sample\;\left[\frac{mg}{g\;protein}\right]}{Essential\;amino\;acid\;pattern\;concentration\;\left[\frac{mg}{g\;protein}\right]\;}\times 100$$

### 2.5. Technofunctional Properties of Optimized LPI

#### 2.5.1. Water and Oil Absorption Capacity

For the water and oil absorption capacity measurement, 0.5 g of LPI was weighed and mixed with 5 mL of water or oil. The mixture was vortexed for 1 min and left to settle for 30 min at room temperature. Then, samples were centrifuged (1600× *g* for 25 min; Beckman Coulter, model Allegra X-22R, rotor SX4250), and the supernatant was discarded. The sample was then weighted again, and the water or oil absorbed was expressed as g oil or water/g LPI.

#### 2.5.2. Foam Properties

Foam properties were evaluated according to the procedure described by Liang et al. [19] with modifications. In total, 1 g of LPI was dispersed in 100 mL of distilled water, and the pH was adjusted to pH 7. The solution was magnetically stirred for 1 h. Then, this solution (*V*_l_ = 100 mL) was placed in a 250 mL graduated cylinder and homogenized using an UltraTurrax disperser (17,500 rpm for 2 min). The foam volume was recorded after homogenization (*V*_0_) and after 30 min (*V*_30_). The foam capacity and foam stability were calculated using the following equations.

(3) $$Foaming\;capacity\;\left(\%\right)=\frac{V_{0}}{V_{l}}\times 100$$

(4) $$Foaming\;stability\;\left(\%\right)=\frac{V_{30}}{V_{0}}\times 100$$

#### 2.5.3. Emulsifying Properties

Emulsifying properties were assessed using the protocol described by Zhao et al. [20], with modifications. In total, 15 mL of LPI solution (1%) at pH 7 was placed in a 50 mL volume falcon tube, and homogenized for 15 s (UltraTurrax, 12,000 rpm). Then, 15 mL of soybean oil were added slowly, and the mixture was homogenized again for 1 min. The emulsion was centrifuged at 1300× *g* for 5 min (Beckman Coulter, model Allegra X-22R, rotor SX4250) and at room temperature. The emulsifying capacity was calculated according to the next equation.

(5) $$Emulsifying\;capacity\;\left(\%\right)=\frac{Volume\;of\;the\;emulsified\;layer\;after\;centrifugation}{Volume\;of\;emulsion\;before\;centrifugation}\times 100\;$$

For the determination of emulsifying stability, the emulsion obtained after homogenization was heated for 30 min at 80 °C. Then, they were cooled at room temperature and centrifuged (1300× *g* for 5 min; Beckman Coulter, model Allegra X-22R, rotor SX4250), and the emulsifying stability was calculated according to the next equation.

(6) $$Emulsifying\;stability\;\left(\%\right)=\frac{Volume\;of\;the\;emulsified\;layer\;after\;heating}{Volume\;of\;emulsion\;before\;centrifugation}\times 100\;$$

#### 2.5.4. Protein Solubility

The LPI solubility (%) was determined over the pH range of 3–9 following the procedure described by Vogelsang-O’Dwyer [21] with modifications. For the measurement, 0.75 g of LPI was mixed with 25 mL of distilled water. The pH of each solution was adjusted using 1M NaOH or 1M HCl, and the suspension was magnetically stirred at room temperature for 1 h. Then, the suspensions were centrifuged (10,000× *g* for 15 min), and 5 mL of the supernatant was used for the nitrogen determination using the Kjeldahl method [12]. The protein solubility was calculated according to the following equation.

(7) $$Protein\;solubility\;\left(\%\right)=\frac{Volume\;\left[mL\right]\times protein\;content\;\left[\frac{g}{mL}\right]}{Sample\;weight\;\left[g\right]\times purity\;\left[\frac{g\;protein}{g\;LPI}\right]}\times 100$$

#### 2.5.5. Gelling Capacity

Gelling capacity determination was based on the procedure described by Lqari et al. [22] with modifications. LPI suspensions between 2% and 20% were prepared in 5 mL phosphate buffer (50 mM; pH 7) at room temperature. The solutions were vortexed for 30 s, rest for 5 min, and vortexed again for another 30 s. The tubes containing the suspensions were heated in a bath (100 °C) for 1 h. After that, the tubes were cooled in an ice bath (1.5 h). Finally, the tubes are inverted, and the gelling concentration is considered as the minimum percentage of LPI necessary to achieve gelling of the sample (sample which did not fall out of or slip from the test tube).

### 2.6. Statistical Analysis

The software Statistics V8.0 (Statsoft Inc., Tulsa, OK, USA) was used to analyze the results, calculate the regression coefficients and optimize the conditions of all responses. The adequacy of the model was determined by the coefficient of determination (R^2^). SPSS software (version 25.0, SPSS Inc., Chicago, IL, USA) was used to analyze the data from lupin flour and LPI characterization using one-way analysis of variance (ANOVA), and significant differences were considered at 5% significance level (*p* < 0.05). The results were presented as mean and standard deviation.

## 3. Results and Discussion

### 3.1. Experimental Design Summary

**Figure 2:**
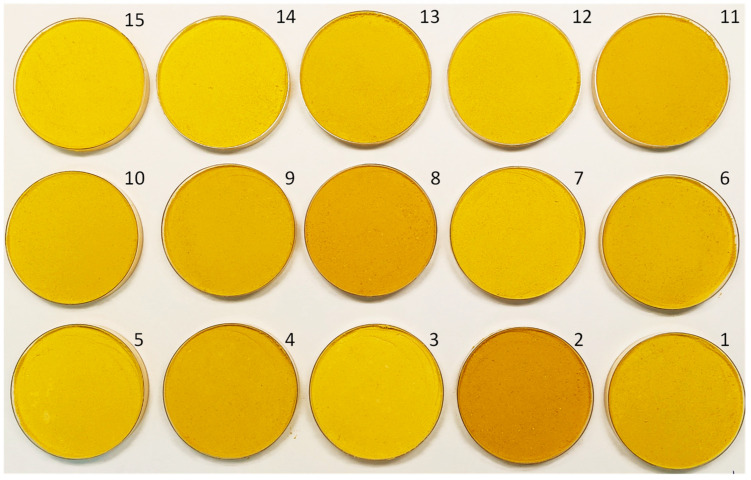
Visual aspect of the different LPI obtained during BBD runs.

As reported in the material and methods section, the Box–Behnken design was used to optimize the conditions to maximize the yield and purity of lupin protein isolates (LPI). A total of 15 runs (with 3 center points) were performed. The experimental results obtained are shown in Table 1, and Figure 2 shows the visual aspect of LPI derived from the Box–Behnken experimental design, which is used to optimize LPI yield and purity.

The yield values ranged from 16.83 to 25.99 g LPI/100 g of lupin flour, while the LPI purity ranged from 78.43 to 89 g protein/100 g of LPI. Based on these experimental values, the regression models have been developed in order to determine the functional relationship for approximation and prediction of responses, and regression coefficients obtained from the ANOVA test are shown in Table 2. The determination coefficients showed a high model accuracy (R^2^ = 0.82532 for yield and R^2^ = 0.93525 for purity), which suggests a strong correlation between predicted and experimental data.

**Figure 3:**
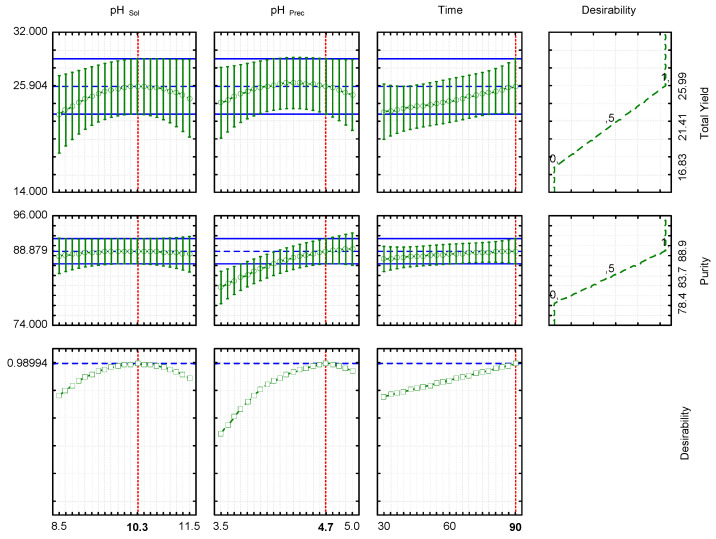
Profiles for predicted values and desirability.

The yield is an important parameter since it is vital to extract the maximum amount of protein possible from the flour. The highest yield values were obtained in the intermediate pH values (pH _Prec_ = 4.32 and pH _Sol_ = 10.3), which also increase with the extraction time (maximum value at 90 min) (Figure 3). However, it is important to highlight that the statistical analysis showed that independent variables did not produce a significant influence on the yield.

Not only is the total yield important, but also the purity of the obtained LPI. That is, the protein concentration of the isolate must be as high as possible, ensuring greater purity. In this case, the linear term of the solubilization pH and the precipitation pH had a significant influence on purity. According to the results (Table 2), the linear term of pH _Sol_ and pH _Prec_ had a positive impact on the LPI purity, and also the interaction between pH _Sol_ and pH _Prec_, while the quadratic terms had a negative effect. The highest purity values were obtained at pH _Sol_ 10.15 and pH _Prec_ 5 (Figure 3). The extraction time did not show significant differences, but the highest values for purity were also obtained after 90 min of extraction. It is clear that pH _Sol_ and pH _Prec_ had an important influence on the purity, while both, yield and purity increased as increase extraction time (*p* > 0.05).

**Figure 4:**
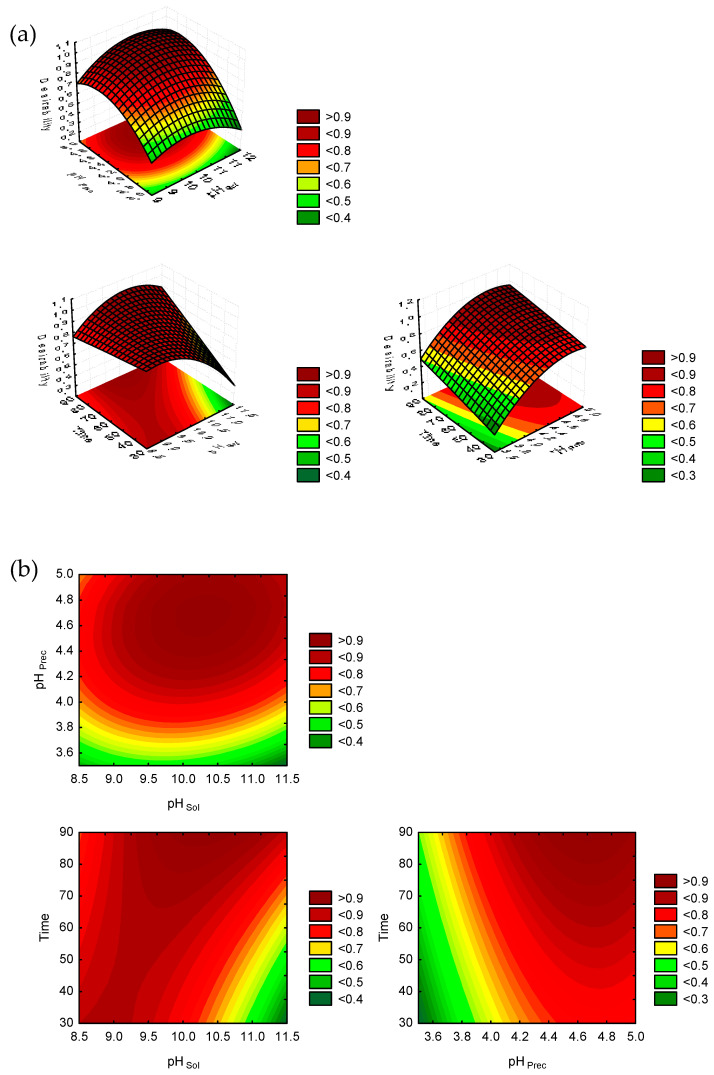
Response surface plots (desirability) (**a**) and Response contour plots (desirability) (**b**) as function of pH _sol_, pH _Prec_ and extraction time.

The optimal operating conditions were calculated through a simultaneous optimization technique called Response Desirability. During desirability determination, all independent variables were maximized. The desirability surface plot and contour plot are shown in Figure 4a and Figure 4b, respectively. According to this technique, the values of the independent variables that maximize both LPI yield and purity were pH _Sol_ 10.3, pH _Prec_ 4.7, and the time was 90 min. The predicted values of the responses for optimization based on higher desirability were 25.9 g LPI/100 g flour for yield and 88.87 g protein/100 g LPI for purity.

The accuracy of the response surface model developed for prediction was established by comparing the predicted values and the experimental results (Table 3). Four independent extractions were carried out in the optimal conditions, and the yield and purity were analyzed. Experimental values showed that the real yield in the optimal conditions was 23.19 g LPI/100 g of flour, and purity was 87.74 g protein/100 g LPI.

As can be seen, experimental data were within the predicted values range, and the mean values were very similar to the predicted values. For yield, the %RSD was 7.78%, while for purity, this value was 0.91%. Thus, this model was a good tool for optimizing the protein extraction process.

### 3.2. Characterization of Lupin Flour and Optimized Lupin Protein Isolate

#### 3.2.1. Chemical Composition and Color Parameters

Taking into account the initial protein content of the lupin flour (42% of DM), the yield obtained during the protein isolate preparation (23.19 g LPI/100 g of flour), and its purity (87.74 g protein/100 g LPI), it can be deduced that 48.45% of the total extractable proteins have been extracted. This value was slightly higher than those described by Albe-Slabi et al. [3], who found that lupin protein extractability was 41–43% between pH 7 and 10. This fact coincides with the solubility of lupin proteins. Extractability is expected to be higher the further the pH is from the isoelectric point (4.7), while the minimum extractability should be at a pH close to the isoelectric point. Similar protein yields were obtained in another study (about 40%) where the authors reported that approximately 60% of protein remained undissolved within lupin flour during extraction [23].

The chemical composition of lupin flour and LPI is shown in Table 4. The moisture of lupin flour was 8.14 g/100 g, which was similar to those reported in *L. mutabilis* seeds (11.7%) [1].

The lipids in lupin flour were 6.06 g/100 g. This data is in line with the typical oil content in *Lupinus Luteus* L. (4.5–6%) [2,24], although other authors reported higher oil values in *L. angustifolius* (13.6%) [22], *L. albus* (10.4–12.6%), or *L. mutabilis* (13–25%) [1,2]. In a recent study, the authors also reported that *L. luteus* presented lower lipid content (4.6%) than other lupin species (between 6.8 and 14.07%) [25]. As previously mentioned, the fact that having a low lipid content is an advantage, it is inferred that lipids may be less in the extraction of proteins. In our particular case, where there is no defatting phase, this aspect is even more important to obtain a good quality protein isolate. In the present study, during the LPI preparation, the lipid content was concentrated (8.87 g/100 g in LPI vs. 6.06 g/100 g in flour). This fact agrees with those reported by other authors, who observed that lupin oil is present during aqueous fractionation, and thus also in the final LPI [26]. In a recent study, the authors also observed higher lipid content in LPI obtained from full-fat *L. albus* and *L. angustifolius* than in the lupin flours [10]. Moreover, the addition of NaOH during alkaline extraction lead to the saponification of lipid component, which increases the “emulsification” of lipid into the aqueous phase and contributes to increasing lipid content in the protein isolate [4]. This fact can explain the higher lipid content in LPI than in lupin flour obtained in the present study. In contrast to us, other studies described lower values (~0.5–2%) of lipids in lupin protein isolates [24,27], while others reported values between 10.6 and 17.04% of fat in protein isolates obtained from *L. albus* and *L. angustifolius* [10].

The protein content in lupin flour was 42 g/100 g, while after isolate preparation, this value (LPI purity) increased to 87.74 g/100 g LPI. It is well known that the protein represents about 29–53% of lupin [2,28], but in *L. luteus*, the protein values ranged from 44.77 to 48.2% [2], which agree with our values. Similar protein values (44.7%) were also reported in *L. mutabilis* seeds [1], *L. albus* flour (43.1%), and *L. angustifolius* flour (41%) [10]. Obviously, after the protein extraction process, the LPI has a much higher content of this macronutrient than flour. The protein content in LPI was also higher than those reported by other authors in a recent study, in which the lupin protein isolate presented a purity between 66.5 and 75.8% [3], while others obtained similar values (87–90%) [29]. LPI obtained from *L. albus* (83.96–94.4%) [21,27,30], defatted *L. campestris* (93.2%) [31], or *L. angustifolius* (81.2–92.6%) [10,21,30] also have similar values to those found by us in protein isolate of *L. luteus*.

The ash content was 4.05 g/100 g in lupin four and 3.18 g/100 g in LPI. Similar values of ash were described previously in *L. luteus* (4.3–5.1%) and in other lupin species (3.4–5%) [2,24]. Multiple protein isolates from *L. albus* and *L. angustifolius* has between 3.14 and 4.01% of ash [27,30], which perfectly agree with values obtained in our LPI, while in LPI from *L. luteus* had lower ash content (1.42%) [24]. As occurs in our study, Muranyi et al. [32] and Piornos et al. [24] reported a higher ash content in flour than in LPI. This could be related to the fact that concentrating the protein results in a decrease in the contribution that the ashes have to the total dry matter. In fact, the fiber contained in the flour undoubtedly contributes an important part of the ashes, but during the preparation of the LPI, it is eliminated, which means that since it is not present in the LPI, the ash content also decreases.

Color parameters showed that both, lupin flour and LPI presented a light-yellow color. LPI had lower L* values, while a* and b* were higher than lupin flour (Table 4). Thus, the increase of redness and yellowness coordinates implies that LPI had a higher orange tone than flour. This fact agrees with results reported by other authors, who concluded that LPI presented a clear yellow color [3]. Additionally, the appearance of the LPI obtained in this study (Figure 2) is the same as those found by other authors [32]. It is well known that lupins contain pigments such as carotenoids [6]. In fact, in a recent study, the authors found significant carotenoids content in lupin seeds (6.12–65.52 mg/kg), in which lutein (orange-red pigment) was the major (70–90%) compound [33]. Carotenoids are lipid-soluble compounds, and the fact that LPI had a higher lipid content than lupin flour implies that LPI also presented a higher amount of these compounds. Therefore, this explains the higher a* and b* values in LPI than in lupin flour.

#### 3.2.2. Fatty Acid Profile

Lupin oil is rich in unsaturated fatty acids [29]. This fact agrees with the results obtained in the present study, in which monounsaturated fatty acids (MUFA) and polyunsaturated fatty acids (PUFA) of oil extracted from lupin flour and LPI were the major fatty acids, representing each about 40% of total fatty acids (Table 5). The content of saturated fatty acids was 22.61 g/100 g in lupin flour and 20.79 g/100 g in LPI, which agree with the results published by other authors on lupin [1].

The main fatty acids in both lupin flour and LPI were C18:1n-9 (~35 g/100 g) and C18:2n-6 (32 g/100 g), followed by similar contents of C16:0, C22:0, and C18:3n-3 (ranged between 5.2 and 7.85 g/100 g) (Table 5). These five fatty acids represent more than 85% of the total fatty acids in lupin oil. The same fatty acid profile was recently found in the *L. mutabilis* seed [1]. However, these authors reported higher amounts of C16:0, C18:1n-9, and C18:2n-6, and lower values of C22:0 and C18:3n-3 than those found by us. In contrast, although the profile was similar, the content of C18:1n-9 in *L. albus* and *L. mutabilis* was higher (56–60%) and the C18:2n-6 was lower (18–26%) [34] than those found in *L. luteus* in the present study. The same authors observed in *L. albus* that the contents of C16:0 and C18:3n-3 were very similar to our results (6–8%), but in *L. mutabilis*, C16:0 had higher values (8.2%) and C18:3n-3 (2.5–2.8%) lower values than in the current research. Finally, the proportion of C22:0 ranged between 0.7 (*L. mutabilis*) and 3.5% (*L. albus*), values lower than those obtained by us in *L. luteus*. In a recent study, the authors analyzed the fatty acid composition of several cultivars of *L. angustifolius*, *L. albus,* and *L. luteus* [33]. Generally speaking, *L. albus* presented the highest amounts of MUFA (>60%) and the lowest of PUFA (~20%), *L. luteus* had the highest PUFA content (~60%) and the lowest of MUFA (26%), while *L. angustifolius* had intermediate values of MUFA (36–47%) and PUFA (31–44%) [33]. In all cases, the SFA represented between 12.51% and 21.7%. Therefore, it seems clear that fatty acids vary significantly between different lupin species. In addition, there are also significant variations between cultivars, which would explain the differences found between the studies.

In our study, there are multiple significant differences between lupin flour and LPI fatty acids. For fatty acids which represent more than 1% of total fatty acids, LPI had higher amounts of C16:0, C18:1n-9, and C18:1n-7, while lower content of C18:3n-3, C20:0, C20:1n-9, C22:0, and C22:1n-9 in comparison with lupin flour. These differences determine that the content of SFA and PUFA was also lower and the content of MUFA was higher in LPI than in lupin flour. Despite these significant differences, it is important to highlight that the fatty acid that presented the greatest variation was C18:1n-9, and this variation between LPI and flour was only 2.6%. Therefore, it can be affirmed that although the extraction did slightly modify the content of some fatty acids, the profile and quality of the lupin oil are practically the same in the lupin flour as in the LPI.

Thus, taking into account the relatively low SFA content, and the high amounts of MUFA (in particular C18:1n-9) and PUFA (C18:2n-6 and C18:3n-3), it can be concluded that the oil from *L. luteus* flour and LPI had a healthy profile. The n-6/n-3 ratio in both cases was ~5, which was close to the value considered “healthy” (4). This result agrees with the n-6/n-3 value obtained in multiple lupin species, which range between 2.14 and 7.96 [33]. With all these in mind, the oil obtained from *L. luteus* could be beneficial to reduce cholesterol and reduce the risk factors associated with heart disease [35]. The same conclusion was obtained for other authors in *L. mutabilis* [34].

#### 3.2.3. Amino Acids Content and Chemical Score

The deficit of valuable proteins is a current problem in the world [1]. Thus, the complete characterization of the amino acid composition and their quality (chemical score) is vital to know the suitability for human nutrition.

Table 6 shows the amino acid content of lupin flour and LPI. In both cases, the major amino acid was glutamic acid (~230 mg/g protein), followed by arginine (~120 mg/g protein) and aspartic acid (~100 mg/g protein); however, low amounts of cysteine (13–17 mg/g protein) and methionine (~4 mg/g protein) were found. Exactly the same composition was reported in LPI obtained from *L. albus* and *L. angustifolius* [10,21], in which glutamic acid content ranging about 23–27%, aspartic acid and arginine, with similar values (10–13%), while cysteine represents ~1% and methionine ~0.5%. In the case of Vogelsang-O’Dwyer et al. [21], the amino acid profile and the content of glutamic acid (~230 mg/g protein), arginine (~110 mg/g protein), aspartic acid (~100 mg/g protein), cysteine (~11 mg/g protein) and methionine (~3 mg/g protein) were coincident with our values. Similarly to our findings, Lqari et al. [22] found the same amino acid profile for both lupin flour and LPI from *L. angustifolius*. In a recent review, the authors also reported high amounts of glutamic, arginine, and aspartic acid, while the lowest values were for cysteine and methionine in flour form *L. albus*, *L. angustifolius*, *L. luteus,* and *L. mutabilis* [2]. This fact proves that although some variations can be due to the lupin specie, the amino acid profile did not vary.

The isolation procedure produces significant changes in the amino acid contents. In our case, the proportions of glutamic acid, tyrosine, valine, leucine, isoleucine, and phenylalanine were higher in LPI, while the concentrations of glycine, arginine, alanine, histidine, threonine were lower in LPI than in lupin flour. Muranyi et al. [23] also observed changes in the amino acid contents between lupin flakes and lupin isolates. In addition, the content of sulfur-containing amino acids (methionine, lysine, and cysteine) was significantly lower in LPI than in lupin flour. This agrees with other studies who conclude that the proportion of these sulfur amino acids decreases during isolation [22]. These authors attributed this loss to the elimination of albumins, rich in these amino acids, during the LPI preparation.

During the protein extraction process, some types of proteins are more extractable than others. The lupin proteins are albumins and globulins, but the major storage proteins are globulins (~80–90%) [31], which can be classified into four different groups (α-, β-, γ- and δ-conglutins) [24]. Each type of protein has different solubilities and properties [23]. In fact, as mentioned before, with the proposed procedure and under optimal conditions, 48.45% of the proteins contained in the flour have been extracted, which means that half of the proteins were retained in the pellet. For this reason, and taking into account that there are differences in the constitutive amino acids in each type of protein, it was expected that the amino acid content of the LPI would be different from that of the flour. The same conclusion was reported in a previous study, in which the authors attributed the amino acid differences between *L. campestris* LPI to the different protein fractions extracted, which have distinct amino acid patterns [31]. However, it should be noted that these differences were minor, and despite the differences in the individual contents of some amino acids, the amino acid profile was the same in both cases, which perfectly agrees with the results reported by other authors [22].

The content of essential amino acids in both lupin flour and LPI was high, representing ~32%. These data agree with those reported by Boukid and Pasqualone [2], who conclude that *L. luteus* had the highest essential amino acid values in comparison with the other lupins. In a recent study, the authors also reported that in LPI obtained from *L. albus* and *L. angustifolius*, the total essential amino acids varied between 27 and 33% [10].

In addition to the amino acid composition, the nutritional quality of the protein of LPI was also evaluated. The mean values of the chemical score, as proposed by FAO/WHO/UNU [18] for humans (adults) are shown in Table 7. The LPI obtained in the present study had amino acid values in agreement with those reported by FAO/WHO/UNU [18], except for valine and methionine who were limiting, providing 95.26% and 22.27% of requirements, respectively. The low content of methionine also determines that the chemical score of sulfur-containing amino acids (methionine + cysteine) was limiting (79.14%), as reported previously by Chukwuejim et al. [4]. Thus, except for these amino acids, the LPI obtained in the present study satisfied the FAO requirements for the essential amino acids [18]. Our results perfectly agree with those reported by Vogelsang-O’Dwyer et al. [21], who conclude that LPI from *L. albus* and *L. angustifolius* were deficient in methionine, valine, and sulfur-containing amino acids. Other authors found that LPI from *L. albus* and *L. angustifolius* were also deficient in lysine [21,22], while our results demonstrated that the LPI of *L. luteus* presented a good chemical score for this amino acid (112.38%). The rest of the chemical scores were between 137.23% and 230.79%, which agrees with other studies [21]. The results obtained in the present study agree with those reported by several authors, who conclude that lupin protein contains low amounts of sulfur-containing amino acids and high lysine content [9].

### 3.3. Technofunctional Properties of Optimized LPI

The technofunctional properties of lupin protein isolate include water and oil absorption capacity, foam and emulsion capacity and stability, gelling capacity, and protein solubility. These properties are directly dependent on the isolation procedure and the extraction conditions. This is due to any irreversible change in protein during LPI preparation, which lead to the protein unfolding and losing functionality. Additionally, some properties are related to specific lupin proteins. In this sense, α- and β-conglutins have excellent emulsification properties, while δ-conglutins have good solubility and foaming capacity [2].

#### 3.3.1. Water and Oil Absorption Capacity

Water and oil absorption capacity are two important attributes, since in food formulation these properties can affect texture, flavor, or mouthfeel [4]. The water absorption capacity depends on the availability of polar amino acids for protein–water interactions, while the oil absorption capacity could be related to the protein flexibility, which determines that proteins are able to expose hydrophobic groups to oil [36]. Therefore, these parameters are highly dependent on the protein denaturation.

In our case, the water absorption capacity was 1.41 g/g, while the oil absorption capacity was 1.66 g/g (Table 8). *L. albus* protein isolate presented also similar values for water (0.8 mL/g) and oil (1–1.3 mL/g) binding capacity [27]. In LPI from *L. angustifolius,* the water and oil absorptions capacity was in both cases 0.85 mL/g [36], and *L. luteus* LPI presented very similar values of water absorption (1.68 mL/g) and oil absorption ability (1.43 g/g) [24]. Similarly to our findings, the LPI obtained from *L. campestris* had 1.7 mL/g for water and oil absorption capacity [31]. Other authors reported higher values for both parameters. For example, in LPI obtained from *L. albus* and *L. angustifolius*, Kebede and Teferra [30] observed that water absorption was 2.7 g/g, while oil absorption capacity was 2.5–2.6 g/g. In another study, the lupin (*L. angustifolius*) protein isolate had 4.46 g/g for water and 1.95 g/g for oil absorption capacity [22].

#### 3.3.2. Protein Solubility

**Figure 5:**
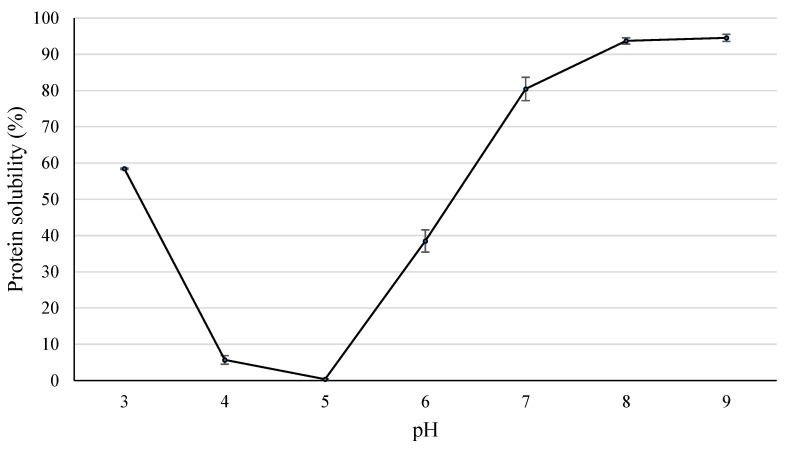
Protein solubility of LPI at pH range of pH 3 and pH 9.

The protein solubility of the LPI as a function of pH ranging between 3 and 9 is shown in Figure 5. Protein solubility is a vital attribute with special relevance to other technofunctional properties (gelling, foaming, emulsifying, etc.) and for food applications [4,9]. As can be seen, the typical U-shaped curve showed the minimum protein solubility at pH 5 (0.39%), and low values were between pH 4 and 5 (<6%). The same results were reported for LPI obtained from *L. angustifolius* [36] and from *L. luteus* [24]. These results are expected since these pH values are close to the isoelectric point (4.7). When the pH value decreases (pH 3) or increases (pH >6), the protein solubility increased dramatically and progressively. It is well known that lupin protein is soluble at strong acidic and alkaline pH [3]. At pH 3, the protein solubility reached 58.4%, at pH 6 solubility was 38.5%, and at pH 7, solubility achieved 80.4%. The highest protein solubility was obtained at pH 8 and 9, with similar values (93.7% and 94.5%, respectively). The explanation for this behavior is that at alkaline pH, the negatively charged proteins exhibit a strong repulsion which favors protein solubilization [9]. At acidic pH, the high solubilization is also related to the repulsion forces, due to proteins being positively charged. Our values were slightly lower than those reported by Albe-Slabi et al. [3] for lupin protein isolate extracted at pH 7, but higher than those obtained through acidic extraction. At pH 7, our values were higher (80.4%) than protein solubility of *L. albus* isolate (64–76.9%) [21,27] and *L. angustifolius* (~70%) [21,36,37]. It is important to highlight that in the present study, no solvents or heat treatment was used, thus no denaturation of proteins was promoted, while in other studies, the procedures which denature protein result in LPI with lower protein solubility [28]. Moreover, our values of protein solubility at pH 3, 5, and 6 were very similar to those reported in isoelectric precipitation LPI obtained from *L. angustifolius* [36]. These authors also reported that LPI obtained from micellization exhibits higher solubility than those obtained by alkaline solubilization-isoelectric precipitation. This fact could be related to micellization producing less protein damage, while isoelectric precipitation increases protein denaturation [9].

**Figure 6:**
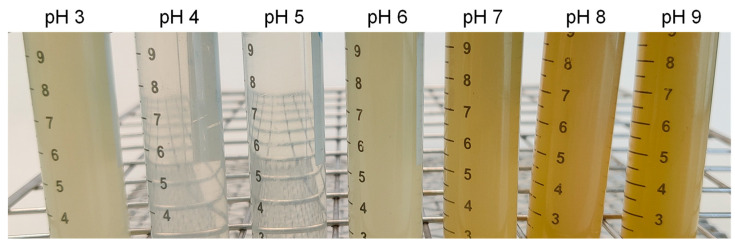
Visual appearance of the LPI solutions at different pH (after removing the insoluble fraction by centrifugation).

Therefore, the protein solubility is highly dependent on the protein unfold ability. At the isoelectric point, the net charge in proteins is minimal [24], and thus, the repulsive forces and flexibility to unfold are low. In the isoelectric point, there are low protein–protein interactive forces, which cause protein aggregation, precipitation, and reduce solubility [28]. However, as the pH moves away from the isoelectric point, the charges increase, favoring the proteins unfolding, repulsive forces, and their solubility. In summary, and in accordance with other authors [4,9,24], the lowest values for protein solubility was at pH 4–5, while the highest values were at pH 8–9. The visual appearance of solubilized protein could be appreciated in Figure 6.

#### 3.3.3. Foam Capacity and Stability

Foaming is another important technofunctional property related to the ability of LPI to create stable air bubbles, which influences several physicochemical properties of food products [28]. Table 8 shows the foam capacity and stability of optimized LPI. According to the results, LPI had 135.3% of foam capacity. This value was lower than those reported in another recent study (212–242%) [3], although it is important to highlight that these authors used a phosphate buffer for the foaming capacity measurement. Similarly, the lupin protein isolate (isoelectric precipitation) of *Lupinus campestris* also presented higher values (about 220%) of foam capacity at pH 6 and 8 than those observed by us. However, in line with the results obtained in the present research, other authors reported values of foam capacity of 119% in lupin protein isolate [22] and 112% in soy protein isolates [20]. In contrast, others reported very low foam capacity to LPI obtained from *L. albus* (31.86–60%), *L. angustifolius* (49.28–60%) [21,30] and *L. luteus* (89.29%) [24]. In view of the results, and in agreement with other authors [28], LPI presented low-to-moderate foaming capacity and stability.

Foaming stability was 76.9% after 30 min. In line with our results, foam stability of LPI obtained by isoelectric precipitation and ultrafiltration ranged between 40 and 70% [3], while LPI for isoelectric precipitation, in another study, had higher foam stability (96%) after 40 min [22]. Similarly, in 1% solutions of LPI obtained from *L. albus* and *L. angustifolius*, the foam stability after 1 h was about 90% [21]. In LPI obtained from *L. luteus*, the foam stability was lower (43.91%) than those obtained by us, but it is important to highlight that these authors measured the foam stability after 2 h [24], which can explain the differences between both studies.

It is well known that the foam capacity and stability are related to the protein molecular properties. The foam capacity is related to the diffusion of proteins between the air-water interface, which stabilize the gas bubble, while protein stability involves the formation of a thick, cohesive, and viscoelastic film around the bubble [31]. Therefore, other functional properties, such as solubility, are vital to improve the foam’s capacity and stability. Additionally, foaming capability can be improved with increasing protein concentration [21]. The foam capacity also increases as increase pH [24]. This is related to at alkaline pH the protein solubility increase, which also increase protein concentration and foam capacity [38].

#### 3.3.4. Emulsion Capacity and Stability

The amphiphilic character of proteins makes them excellent candidates for emulsion formation. Thus, these proteins are adsorbed at the oil-water interface, decreasing surface tension and stabilizing emulsions [4]. Consequently, the LPI is postulated as a potential emulsifier to be used in the food industry [9]. In the present study, the emulsion capacity of LPI obtained in the optimized conditions was 60.6%, while emulsion stability was 55.4% (Table 8). These values are in the range reported by other authors for LPI (74.5% capacity and 71% stability) [22], and soy protein isolate (50.94% capacity and 51.22 stability) [20]. In a recent study, the authors found that the emulsion capacity of LPI obtained from *L. albus* and *L. angustifolius* was significantly lower than those obtained in the present study (~49%) [30]. Thus, in the present work, the emulsion capacity and stability were similar or higher to those found in other protein isolates.

The emulsion capacity is highly dependent on the protein behavior. When the protein of LPI is unfolded, there are exposed more hydrophobic groups, which can be associated with lipid fraction and increase emulsion capacity and stability [38]. Moreover, these authors also conclude that the pH is not a critical parameter in relation to the LPI emulsion properties.

#### 3.3.5. Gelling Capacity

The gelling capacity is a vital feature for several foods due to gelling agents are necessary to achieve the required texture or consistency. Gelling ability of protein isolates varies with several factors, including protein extraction procedure or lupin species. Thermal treatment unfolded the proteins, resulting in more interactions between exposed groups and forming a continuous protein network [4]. The gelling ability of lupin protein isolates was related to their resistance to thermal unfolding, which result in a weaker gel [2]. Thus, the heating treatment produces partial protein denaturation, which retained more water amount into the gel structure, and transforms the liquid into a gel [24]. Accordingly, gelation ability is a mixture of chemical changes, which include protein denaturation, aggregation, and network formation [9]. In the present study, the gelling capacity of LPI was 10.3% (Table 8). This value agrees with those reported previously for protein isolates of *L. angustifolius*, which range between 10 and 12% [22]. In contrast, other authors reported in LPI from *L. luteus* gelling capacity of 20% [24], but these values ranged between 14 and 20% depending on the pH. In a recent study, the authors proved that the lupin species is vital in the gelling capacity [21]. These authors observed that gelling capacity of LPI obtained from *L. albus* was 7% (similar to our values), but this value in LPI from *L. angustifolius* was 23%. Thus, it seems that *L. angustifolius* has a poorer gelling performance than *L. albus* [21].

## 4. Conclusions

The alkaline solubilization following isoelectric precipitation is the main procedure to obtain protein isolates. However, several factors can affect both, the yield and purity of these isolates. With this in mind, the effect of different parameters, such as the pH and the extraction time was studied in the present research. The solubilization and precipitation pHs were the most important parameters affecting the lupin purity. After the Box–Behnken design, the optimal conditions were at pH _Sol_ = 10.3, pH _Prec_ = 4.7, and extraction for 90 min. The model was a good tool to predict the yield and purity values, and under optimal conditions, the experimental results were 23.19 g LPI/100 g flour for yield and 87.74 g protein/100 g LPI for purity.

The optimized LPI had important content of high-quality proteins. The major amino acids were glutamic, aspartic acid, and arginine. The protein chemical scores showed that the protein isolate was only deficient in methionine and valine. Therefore, LPI protein contains high amounts of essential amino acids, and it would cover the nutritional requirements for humans, following the criteria of the FAO. Similarly, the fatty acids composition showed that lupin oil had important amounts of healthy unsaturated fatty acids.

On the other hand, the optimized LPI showed interesting technofunctional properties, such as foaming or emulsifying capacity and stability, oil and water absorption capacity, and gelling capacity. Additionally, the protein solubility was >90% at pH 8 or higher. However, the technofunctional properties of LPI are lower than those of other protein isolates.

Therefore, in view of these results, it can be affirmed that lupin protein isolates can constitute an ingredient or an agent for the formulation of the food, with excellent nutritional composition, protein quality, and adequate properties similar to those of the protein isolates currently used. For this reason, it can be concluded that the use of LPI by the food industry would allow the development of fortified foods (with a higher content of high-value proteins, or peptides with health benefits) at the same time that it would serve to improve the food properties (gelling, foaming, emulsifying capacity, etc.).

Further studies must be carried out to fully characterize the isolates, as well as to propose alternative and clean technologies in order to increase the purity and/or improve the technofunctional properties of the isolate obtained in this study. These studies are necessary to valorize the lupin protein isolate for their incorporation into the human diet.

## Figures and Tables

**Table 1:** Box–Behnken design (natural and coded values) of extraction conditions and experimental results obtained for dependent variables.

	pH _Sol_	Time	pH _Prec_	Yield	Purity
	Independent variables			Dependent variables	
	X_1_	X_2_	x_3_	y_1_	y_2_
1	8.5 (−1)	60 (0)	3.5 (−1)	20.70	81.86
2	11.5 (1)	60 (0)	3.5 (−1)	18.68	78.43
3	8.5 (−1)	60 (0)	5 (1)	22.05	89.00
4	11.5 (1)	60 (0)	5 (1)	21.50	88.60
5	8.5 (−1)	30 (−1)	4.25 (0)	23.31	88.38
6	11.5 (1)	30 (−1)	4.25 (0)	16.83	81.36
7	8.5 (−1)	90 (1)	4.25 (0)	25.01	88.11
8	11.5 (1)	90 (1)	4.25 (0)	25.53	86.27
9	10 (0)	30 (−1)	3.5 (−1)	21.34	82.27
10	10 (0)	30 (−1)	5 (1)	24.26	88.15
11	10 (0)	90 (1)	3.5 (−1)	23.01	81.86
12	10 (0)	90 (1)	5 (1)	23.56	87.90
13	10 (0)	60 (0)	4.25 (0)	25.99	87.19
14	10 (0)	60 (0)	4.25 (0)	23.64	87.03
15	10 (0)	60 (0)	4.25 (0)	24.26	86.75

Yield: g LPI/100 g flour; Purity: g protein/100 g LPI.

**Table 2:** Regression coefficients of the second-order polynomial equation and statistical parameters.

	Yield		Purity	
	Regression Coefficient	*p* Values	Regression Coefficient	*p* Values
Mean/Interc. (β_0_)	−96.7433	0.000000	31.16198	0.000000
Linear				
pH _Sol_ (β_1_)	14.5347	0.148883	1.15322	0.026705
pH _Prec_ (β_2_)	26.2173	0.187105	24.50919	0.000830
Time (β_3_)	−0.2530	0.071972	−0.25280	0.374784
Crossed				
(β_12_)	0.3262	0.695144	0.67344	0.342223
(β_13_)	0.0389	0.104668	0.02876	0.133128
(β_23_)	−0.0263	0.532150	0.00181	0.957323
Quadratic				
pH _Sol_ (β_11_)	−0.9482	0.068086	−0.33991	0.355657
pH _Prec_ (β_22_)	−3.1325	0.113531	−3.11541	0.067116
Time (β_33_)	0.0002	0.854654	−0.00022	0.806358
R^2^	0.82532		0.93525	

Yield: g LPI/100 g flour; Purity: g protein/100 g LPI.

**Table 3:** The response of predicted and experimental values of the optimized conditions.

Response	Predicted Values	Experimental Values	%RSD
Yield (g LPI/100 g flour)	25.90 ± 3.11	23.19 ± 0.89	7.78
Purity (g protein/100 g LPI)	88.87 ± 2.54	87.74 ± 0.14	0.91

**Table 4:** Chemical composition and color parameters of lupin protein isolate obtained at optimum conditions.

Chemical Composition (g/100 g)	Lupin Flour	LPI	Sig.
Moisture	8.14 ± 0.09	-	***
Lipids †	6.06 ± 0.30	8.87 ± 0.71	***
Protein †	42.00 ± 0.23	87.74 ± 0.16	***
Ash †	4.05 ± 0.03	3.18 ± 0.40	***
Color parameters			
L*	77.59 ± 0.80	74.99 ± 1.14	***
a*	2.76 ± 0.51	4.39 ± 0.07	***
b*	31.92 ± 0.23	45.28 ± 0.23	***

Sig: significance; ***: *p* < 0.001; †: Results expressed as g/100 g of dry matter.

**Table 5:** Fatty acids profile (g/100 g of total fatty acids) of lupin flour and LPI oil.

	Lupin Flour	LPI	Sig.
C14:0	0.25 ± 0.01	0.29 ± 0.01	**
C16:0	6.60 ± 0.02	7.85 ± 0.06	***
C18:0	3.29 ± 0.01	3.32 ± 0.02	ns
C18:1n-9	34.19 ± 0.02	36.83 ± 0.03	***
C18:1n-7	0.65 ± 0.02	0.87 ± 0.03	**
C18:2n-6	32.02 ± 0.01	32.23 ± 0.10	ns
C18:3n-3	6.57 ± 0.01	6.11 ± 0.06	***
C20:0	3.50 ± 0.04	2.77 ± 0.04	***
C20:1n-9	2.25 ± 0.02	1.89 ± 0.01	***
C20:2n-6	0.22 ± 0.01	0.17 ± 0.01	***
C21:0	0.23 ± 0.01	0.18 ± 0.01	***
C22:0	7.20 ± 0.09	5.20 ± 0.08	***
C22:1n-9	1.03 ± 0.01	0.76 ± 0.03	***
C22:2n-6	0.24 ± 0.06	0.18 ± 0.01	ns
C23:0	0.32 ± 0.01	0.21 ± 0.01	***
C24:0	0.99 ± 0.02	0.64 ± 0.03	***
SFA	22.61 ± 0.12	20.79 ± 0.11	***
MUFA	38.26 ± 0.03	40.50 ± 0.04	***
PUFA	39.13 ± 0.10	38.71 ± 0.13	*

SFA: Saturated fatty acids; MUFA: Monounsaturated fatty acids; PUFA: Polyunsaturated fatty acids. In the table only the fatty acids that represented more than 0.1% of the total fatty acids are presented, although all the identified fatty acids have been used for the calculation of SFA, MUFA and PUFA; Sig: significance; ns: not significant; *: *p* < 0.05; **: *p* < 0.01; ***: *p* < 0.001.

**Table 6:** Amino acid composition (mg/g protein) of lupin flour and lupin protein isolate obtained at optimum conditions.

	Lupin Flour	LPI	Sig.
Aspartic acid	101.22 ± 2.86	103.79 ± 3.54	ns
Serine	53.24 ± 0.51	53.36 ± 3.41	ns
Glutamic acid	227.69 ± 5.39	236.09 ± 4.86	**
Glycine	44.85 ± 0.56	40.41 ± 1.22	***
Arginine	128.87 ± 3.27	116.5 ± 2.78	***
Alanine	34.76 ± 0.50	33.09 ± 0.93	**
Proline	45.21 ± 2.09	43.44 ± 1.23	ns
Cysteine	17.59 ± 1.04	13.85 ± 1.10	***
Tyrosine	24.89 ± 1.96	31.39 ± 1.88	***
Non-Essential Aas	679.36 ± 4.15	673.68 ± 5.02	*
Histidine	31.84 ± 0.94	28.92 ± 0.80	***
Threonine	42.99 ± 1.05	37.43 ± 0.46	***
Valine	35.89 ± 0.90	37.15 ± 0.45	**
Methionine	4.12 ± 0.55	3.56 ± 0.31	*
Lysine	56.92 ± 3.28	50.57 ± 1.76	**
Isoleucine	37.97 ± 1.23	43.56 ± 0.85	***
Leucine	71.41 ± 2.11	80.96 ± 2.05	***
Phenylalanine	39.60 ± 2.26	44.16 ± 2.61	**
Essential Aas	320.64 ± 4.15	326.32 ± 5.02	*
Essential/Non-Essential	0.47 ± 0.01	0.48 ± 0.01	ns

Sig: significance; ns: not significant; *: *p* < 0.05; **: *p* < 0.01; ***: *p* < 0.001.

**Table 7:** Chemical score of lupin protein isolate obtained at optimum conditions.

	FAO/WHO/UNU (2007)	LPI	
		Mean	S.D.
Histidine	15	192.81	5.35
Isoleucine	30	145.20	2.83
Leucine	59	137.23	3.47
Lysine	45	112.38	3.91
Met+Cys	22	79.14	5.69
Methionine	16	22.27	1.93
Cysteine	6	230.79	18.32
Phe+Tyr	38	198.80	11.76
Threonine	23	162.73	2.00
Valine	39	95.26	1.16
Total indispensable amino acids	277	371.55	7.22

**Table 8:** Technofunctional properties of the lupin protein isolate obtained at optimum conditions.

	LPI	
Technofunctional Properties	Mean	S.D.
Water absorption capacity (g/g)	1.41	0.03
Oil absorption capacity (g/g)	1.66	0.01
Foam capacity (%)	135.3	21.9
Foam stability (%)	76.9	5.14
Emulsion capacity (%)	60.6	4.39
Emulsion stability (%)	55.4	3.76
Gelling capacity (%)	10.3	0.29

## Data Availability

All data are presented in the manuscript.
